# Numerical study on the energy cascade of pulsatile Newtonian and power-law flow models in an ICA bifurcation

**DOI:** 10.1371/journal.pone.0245775

**Published:** 2021-01-25

**Authors:** Samar A. Mahrous, Nor Azwadi Che Sidik, Khalid M. Saqr

**Affiliations:** 1 Department of Thermo-Fluid Universiti Teknologi Malaysia, Skudai, Malaysia; 2 College of Engineering and Technology, Arab Academy for Science, Technology and Maritime Transport, Alexandria, Egypt; 3 Malaysia–Japan International Institute of Technology (MJIIT), University Teknologi Malaysia Kuala Lumpur, Kuala Lumpur, Malaysia; University of New South Wales, AUSTRALIA

## Abstract

The complex physics and biology underlying intracranial hemodynamics are yet to be fully revealed. A fully resolved direct numerical simulation (DNS) study has been performed to identify the intrinsic flow dynamics in an idealized carotid bifurcation model. To shed the light on the significance of considering blood shear-thinning properties, the power-law model is compared to the commonly used Newtonian viscosity hypothesis. We scrutinize the kinetic energy cascade (KEC) rates in the Fourier domain and the vortex structure of both fluid models and examine the impact of the power-law viscosity model. The flow intrinsically contains coherent structures which has frequencies corresponding to the boundary frequency, which could be associated with the regulation of endothelial cells. From the proposed comparative study, it is found that KEC rates and the vortex-identification are significantly influenced by the shear-thinning blood properties. Conclusively, from the obtained results, it is found that neglecting the non-Newtonian behavior could lead to underestimation of the hemodynamic parameters at low Reynolds number and overestimation of the hemodynamic parameters by increasing the Reynolds number. In addition, we provide physical insight and discussion onto the hemodynamics associated with endothelial dysfunction which plays significant role in the pathogenesis of intracranial aneurysms.

## 1. Introduction

Cerebrovascular diseases can lead to life-threatening conditions [1]. Intracranial aneurysm (IA) is considered among the most dangerous vascular disorders that cause millions of deaths every year all over the world [2, 3]. Intracranial aneurysm, is a cerebrovascular lesion in which, the weak area of the blood vessel usually enlarges [2, 4–6]. It is a bulging that protrudes when the blood vessel wall becomes excessively weak to withstand the hemodynamic forces [7, 8]. Intracranial aneurysms arise along a curvature and at a bifurcation of the parent blood vessel in the circle of Willis [9]. The worst outcome of intracranial aneurysm is its rupture resulting in subarachnoid hemorrhage (SAH) causing a high mortality rate [10–14]. It represents one-quarter of cerebrovascular deaths [15, 16]. According to recent World Health Organization data released in 2017, stroke deaths in Malaysia accounted for 11.31% of total deaths, while in Egypt reached 11.04%. IAs occur in 3% to 5% of the adult population [17], exerted without noticeable symptoms. Stroke is the second leading cause of death after heart diseases. It was found that IA ruptures every 18 minutes in the US [18, 19]. Severe disability or sudden death may occur, depending on the severity of the bleeding. The mortality rate as a result of SAH is approximately 40% to 50% [20].

For the past two decades, Computational Fluid Dynamics (CFD) has been the most prominent research tool for investigating the effect of fluid dynamics in intracranial arteries/aneurysms [21]. Previous CFD researches proved that there are strong connections between the genesis of intracranial aneurysm and hemodynamics (i.e. blood flow dynamics in arteries) [22–25]. Despite the large volume of research that examined the effect of various hemodynamic parameters on the initiation and rupture of intracranial aneurysm, the obtained results were divergent and paradoxical.

Several researches concerned with the relation between hemodynamics and intracranial aneurysm suggest instabilities in blood flow in intracranial aneurysm [26–30]. The Reynolds number in cerebral vessels is in the order of 100, which marks laminar pulsatile artery flow according to the classical theory of hydrodynamics. However, recent high-resolution CFD studies have shown the existence of unstable flow and possible transitional or turbulent flow in some cerebral aneurysms at such low Reynolds number [31, 32], which is unjustifiable by the hydrodynamic stability theory. According to Frösen et al. [8], flow instabilities have strong effects in causing endothelial dysfunction of the blood vessel and the subsequent wall degeneration or remodeling associated with IA formation. Hahn et al. [33] showed that the unstable flow pattern, is related to endothelial cell injury. This progresses to abrasion of the endothelial layer as the aneurysm grows. Such conditions were attributed to what was called abnormal shear stress at the aneurysm walls. Once the endothelial cell was damaged, subsequent remodelling and degradation in the vessel wall may occur. Further research, reported in [34], showed that the endothelial dysfunction and abrasion of the endothelial cells can never happen under laminar flow conditions, the flow must be disturbed. Nevertheless, these results were considered controversial since vascular blood flow occurs at Reynolds number that is one order of magnitude less than the critical value at which the onset of transition occurs in Newtonian fluids. It was found that wall shear stress (WSS) plays an important role in the onset, growth and rupture of IAs as the endothelial cells detect the change in WSS [35]. However, the mechanism by which WSS leads to the initiation, evolution and rupture of IA is not entirely understood. There exists a significant controversy in literature concerning whether low or high WSS is associated with initiation and rupture of IA [32, 36, 37].

Another ongoing controversy in literature is whether Newtonian or non-Newtonian viscosity models should be adopted while modeling blood flow in intracranial arteries and the effect of the blood rheology on the WSS findings [38]. The majority of numerical simulations of intracranial aneurysm, have assumed the blood as Newtonian fluid when solving the Navier-Stokes equation for both the steady and pulsatile flows [7, 22, 39–41]. It is nearly a consensus that the alterations of blood viscosity are inconsiderable. Some of these studies have not discussed the fact of this assumption at all [42–44]. While other studies mentioned that the vessel diameter is large enough to disregard the non-Newtonian behavior of blood [45, 46]. However, several studies highlight the significance of non-Newtonian effects in intracranial blood flow [47–52]. Evju et al. [53] have demonstrated that there are no differences between the Newtonian and the non-Newtonian models on the WSS, while, Xiang et al. [52] have presented that the Newtonian model obviously overestimated the WSS. Suzuki et al. [51] conducted a comparative study on WSS of intracranial aneurysm and found up to 25% differences on normalized WSS between the non-Newtonian and the Newtonian models. Frolov et al. [47] performed in vitro study using Newtonian and non-Newtonian viscosity model to examine the WSS in the internal carotid artery aneurysm. Their results showed that the use of Newtonian model over-predicts WSS by 19.7% at the aneurysm dome. Otani et al. [50] studied the hemodynamics in a coiled IA using two viscosity models. They obviously demonstrated that the use of a non-Newtonian viscosity model produces different hemodynamic parameters than the Newtonian model. More precisely they specified that the Newtonian assumption underestimates the shear rate reduction related to thrombus formation in the coiled aneurysm. On the other hand, Goodarzi et al. [54] found that there were small alterations on the WSS and WSS divergence when studying the near-wall velocity field of three aneurysms by using the generalized Carreau model versus the Newtonian model. They concluded that the non-Newtonian effects on the flow pattern could be neglected.

To that end, it is clear that there is no consensus about the effect of the viscosity model on the WSS and the actual hemodynamics which responsible for aneurysm initiation and rupture. To solve this debate other parameters rather than WSS should be investigated to study the impact of non-Newtonian blood behavior in order to understand the characteristics of pulsatile blood flow in intracranial arteries using different viscosity models. This study will guide the Enothelial Cell mechanotransduction researches by providing detailed quantitative and qualitative flow descriptions and characterizing the hemodynamic patterns that govern endothelial cells mechanobiology which can be used to trace biological factors affecting the initiation of intracranial aneurysm.

This paper is motivated by the lack of theoretical understanding of the transition to turbulence in pulsatile flow in intracranial arteries, particularly with non-Newtonian effects. In the current study the authors highlight the importance of considering the non-Newtonian blood behavior by studying the kinetic energy cascade (KEC) rates and the vortex structure to gain a further understanding of the intrinsic flow dynamics to characterize the blood in an idealized internal carotid artery ICA. This work represents the first parametric study to describe the full spectrum KEC (*f* >10) and the KEC of the coherent structure (*f* < 10) at different locations inside the bifurcation model using the shear-thinning behavior of blood. The objective of the present study is to demonstrate the existence of transitional to turbulence in pulsatile blood flow using the non-Newtonian behavior in an ideal cerebral artery at Reynolds number ≤ 600.

## 2. Materials and methods

The methodology used in the present work is to examine the kinetic energy cascade analysis and the vortex structure with non-Newtonian pulsatile flow to depict the biologically fluid flow pattern in an ideal cerebral blood vessel. As the blood flow is not a homogenous isotropic turbulent flow; hence, the use of Taylor’s hypothesis and Kolmogorov scaling laws are not adequate. Kolmogorov’s theory is an asymptotic theory: it has been shown to work well in the limit of very high Reynolds numbers which is not the case in blood flow in cerebral arteries. One way of describing hydrodynamic instabilities is by using spectral analysis. The spectral analysis depends on studying the flow kinetic energy in the Fourier frequency domain and examining the energy spectra characteristics with tools analogous to the tools used in the theory of turbulence. Consequently, a clear understanding of the intrinsic flow dynamics can be deduced better by looking in its frequency domain behavior [55]. This method has the privilege of dealing with nonlinear effects such as energy cascade. While, Kolmogorov model is inapplicable since the flow is not fully developed turbulent. A Direct Numerical Simulation (DNS) was performed using CFD FLUENT, version (16), which uses the finite volume method for the spatial discretization. A monoharmonic pulsatile velocity profile is applied at the inlet of the artery.

### 2.1 Geometrical model

**Fig 1:**
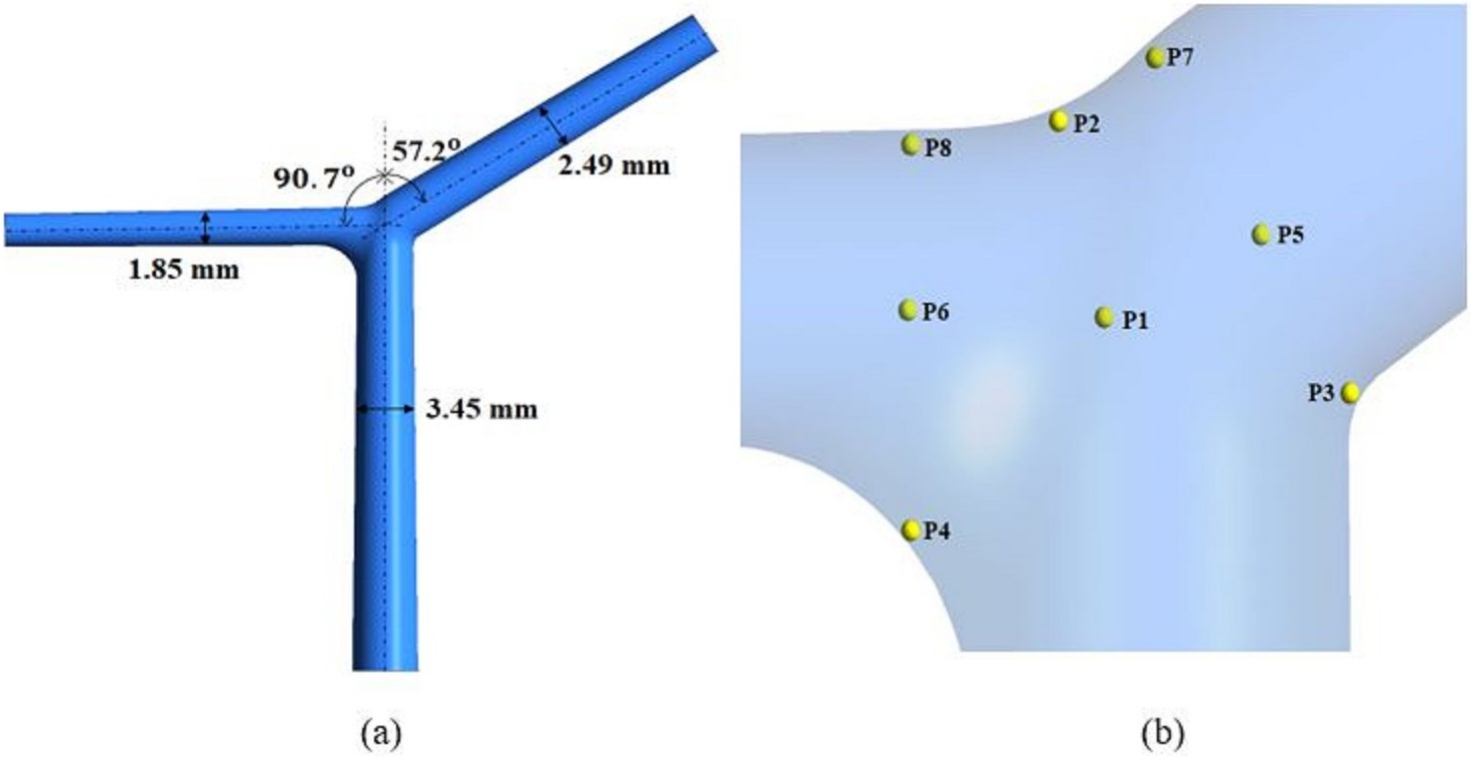
(a) Ideal geometry to model the bifurcation artery with dimension of the parent artery and the daughter vessels (b) zoomed pic to the region of interest with indication of the monitoring points of interest.

An idealized bifurcation model is shown in (Fig 1) is used in order to mimic the intracranial arteries which represent the arteries at the internal carotid artery and this geometry is based on previously published data [56]. The diameter of the parent vessel is 3.45 * 10^−3^ m. The largest daughter vessel (Middle cerebral artery) and smallest daughter vessel (Anterior cerebral artery) are 2.49 * 10^−3^ m and 1.85 * 10^−3^ m in diameter respectively, which correspond to physiological diameters of the major intracranial arteries. The vessel length was set at 20 mm for the parent vessel and 15 mm for the branches.

### 2.2 Governing equations

The blood flow field is assumed to be incompressible, unsteady (pulsatile) and three dimensional (3-D). Thus, the continuity and the Navier-Stokes equations for 3-D incompressible flow are given by:

$$\frac{\partial v}{\partial t}+\nabla .\text{V}=0$$ (1)

$$\rho \left(\frac{\partial \text{v}}{\partial t}+\text{V}.\nabla \text{V}\right)=-\nabla p+\nabla .\tau$$ (2)

Where, V is the velocity vector field of the fluid, *p* is the pressure, *ρ* is the density of the blood 1060 *kg*/*m*^3^ and ***τ*** is the stress tensor.

The stress tensor is related to the strain rate tensor ($\dot{\gamma }$) as follows:

$$\tau =\mu \dot{\gamma }$$ (3)

Where,

$$\dot{\gamma }=(\frac{\partial v_{i}}{\partial x_{j}}+\frac{\partial v_{j}}{\partial x_{i}})$$ (4)

There are several approaches to model the blood flow. In one approach, the blood is approximated as a Newtonian fluid *μ* = 0.0035 Pa.s [39, 56–58]. More refined models, e.g., the power-law model, the Carreau model, the cross model and the Carreau-Yasuda model, include the shear-thinning behavior of blood which capture the non-Newtonian rheology [59–61]. In this study the proposed Power-law model, is used to account for the shear-thinning behavior of the blood [22]. The power-law model offers the simplest representation of shear-thinning behaviour that fit for the intermediate shear-thinning region while other models take into account the existence of a yield stress, as is the case of the Herschel–Bulkley and the Casson models and to simplify the viscosity model that represent the non-Newtonian behavior to prove that with even this simple model, there are significant differences on the hemodynamics parameters when comparing by the Newtonian model. In addition, the power low model is perhaps the most widely used model in literature and has relatively lower computational cost in comparison with other non-Newtonian viscosity models as reported by [47].

$$\mu =k{\dot{\gamma }}^{n-1}$$ (5)

Where, *μ* is the dynamic viscosity, k is the flow consistency index = 0.01467 *Pa*.*s*^*n*^, $\dot{\gamma }$ is the shear rate and *n* is the power law index = 0.7755.

Reynolds number is the only nondimensional parameter required for full dynamic similarity in pulsatile internal flows [62, 63]. Reynolds number which is a dimensionless parameter (the ratio of inertial forces to viscous forces) is used to predict the transition from laminar to turbulent flow. Therefore, the investigation of its effects on the hemodynamics of intracranial arteries is strongly required. The simulations were conducted at Re = 100, 400 and 600 which represent the physiological range of Re in the cerebral arteries [64, 65]. The Reynolds number is calculated based on (6) for the Newtonian viscosity model and (7) for the non-Newtonian viscosity model [66, 67]. Table 1 provides a summary for the corresponding mean velocity at different Reynolds number for the Newtonian and non-Newtonian viscosity models.

**Table 1:** The corresponding mean velocity at different Reynolds number for the Newtonian and non-Newtonian viscosity models for the bifurcation model.

Reynolds number (Re)	*μ*_*m*_ (m/s)	*μ*_*m*_ (m/s)
	Newtonian model	non-Newtonian model
100	0.096	0.168
400	0.383	0.520
600	0.574	0.725

$$Re=\frac{\rho u_{m}d}{\mu }$$ (6)

$$Re_{NN}=\frac{{\rho \text{u}}_{\max^{2-\text{n}}}{\text{d}}^{\text{n}}}{\text{k}}$$ (7)

### 2.3 Boundary condition

**Fig 2:**
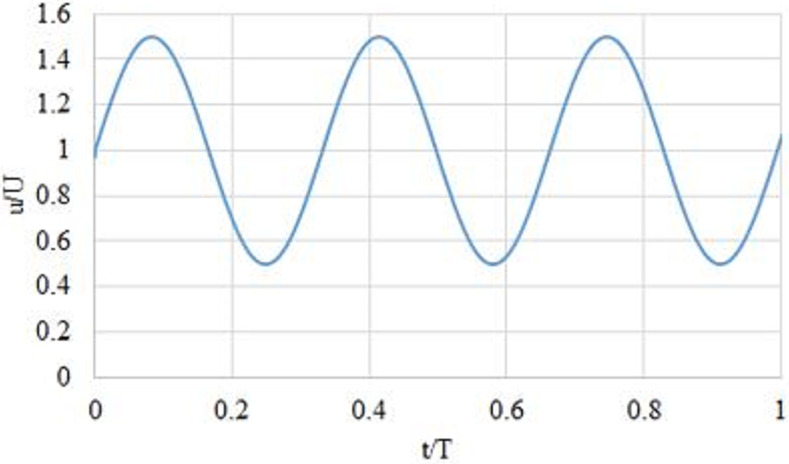
Inlet velocity profile for Newtonian and non-Newtonian fluid models.

A monoharmonic pulsatile velocity profile implemented using user-defined function (UDF) is applied at the inlet and presented at Fig 2, to investigate the kinetic energy cascade rates and the vortex structure of the ICA bifurcation. The boundary condition for the pulsatile Newtonian model has been based on (8) and the boundary condition for the pulsatile non-Newtonian model has been based on (9) [66]. This inlet boundary condition composed of steady (Hagen-Poiseuille solution) component superimposed on an unsteady axial velocity component and zero pressure was specified at the outlet. For the vessel surface, the compliant wall is neglected and hereby employed the non-slip conditions.

Pulsatileinletvelocityprofile=steadycomponent+unsteadycomponent

#### 2.3.1 The boundary condition for the pulsatile Newtonian model

$$u\left(r,t\right)=u_{m}\left[1-\frac{r^{2}}{R^{2}}\right]+u_{os}\;sin\left(2\pi ft\right)$$ (8)

#### 2.3.2 The boundary condition for the pulsatile Power-law model

$$u\left(r,t\right)=u_{m}\left[1-{\left(\frac{r}{R}\right)}^{\frac{n+1}{n}}\right]+u_{os}\;sin\left(2\pi ft\right)$$ (9)

### 2.4 Solution methodology

The blood was considered as an incompressible non-Newtonian fluid controlled by the 3D transient and incompressible Navier-Stokes equations. A Direct Numerical Simulation (DNS) was performed using CFD FLUENT, version (16), which uses the finite volume method for the spatial discretization. The DNS provides the nearest numerical simulation to the analytical solution of the Navier-Stokes equation as it solves all the scales. SIMPLE scheme was used in the simulation for pressure-velocity coupling and second order discretization scheme for pressure and second-order upwind for momentum. The convergence criteria for iterative errors were set to be 10^−6^. Each simulation required approximately 25 days of CPU time on an HPC which is a supercomputer installed at the Universiti Teknologi Malaysia. All the simulations were conducted up to three cardiac cycles so as to get and quantify the transitional characteristics of the flow and the maximal iterations per time step was set 100. An analysis of mesh sensitivity was firstly carried out to make sure the obtained hemodynamic factors are independent of mesh systems at Re = 600. The model was meshed using ICEM-CFD 16.0 software. The mesh refinement was confirmed with several mesh densities with the element number increasing up to 3 million elements based on the velocity profile. Furthermore, the authors confirmed that the intermediate grid with 2 million tetrahedral elements with 10-cells boundary layer have been attached to the wall. This ensures sufficiently high resolution of the velocity fluctuations and capture the near wall flow accurately as no further improvements were observed for finer meshes. The same computational mesh was used both for Newtonian and non-Newtonian cases.

#### 2.4.1. Courant number calculation

The Courant number is a measure of how much information traverses (u) a computational grid cell (Δx) in a given time-step (Δt) and is calculated based on (10).

$$C=\frac{u_{m}\Delta t}{\Delta x}\leq C_{max}$$ (10)

Where,

C is the Courant number, ***μ***_***m***_ is the mean velocity, Δ*t* is the time step of the numerical model and Δ*x* is the spacing of the grid in the numerical model.

The value of *C*_*max*_ varies with the method that used to solve the discretized equation (implicit or explicit). When the explicit method (time-marching) is used then usually *C*_*max*_ = 1.

$$\Delta t=\frac{\Delta x}{u}$$ (11)

The grid size of 2 million elements has Δx = 0.000019036 m and the corresponding time step at different Reynolds number for both rheological models is summarized in Table 2.

**Table 2:** The corresponding time-step at different Reynolds number for the Newtonian and non-Newtonian viscosity models.

Reynolds number (Re)	Δ*t* for the Newtonian model	Δ*t* for the non-Newtonian model
100	2 * 10^−4^ s	10^−4^ s
400	5 * 10^−5^ s	4 * 10^−5^ s
600	3 * 10^−5^ s	2 * 10^−5^ s

## 3. Results and discussion

Hemodynamic parameters in the cerebral arteries play a crucial role in the genesis and growth of numerous cerebrovascular lesions, such as cerebral aneurysms [7, 68, 69]. During the past two decades, computational fluid dynamics (CFD) simulations have been a powerful tool for studying the hemodynamics in intracranial arteries. However, most of these studies assumed the blood as a Newtonian fluid, due to the lack of understanding the influence of non-Newtonian characteristics [70, 71]. The blood flow in large arteries is usually modeled as Newtonian fluid and this hypothesis may be suitable in many cases. However, in other cases, this assumption is not precise, especially when studying the flow in small blood vessels [22, 72, 73]. Additionally, in large arteries at bends and bifurcations the use of Newtonian model is not accurate [74]. In this paper, the authors evaluated the effect of the shear-thinning characteristic on the kinetic energy cascade rates and the vortex structure in the idealized internal carotid bifurcation artery in order to gain a further understanding of the intrinsic flow dynamics.

### 3.1 Spectral analysis

The spectral analysis depends on studying the flow kinetic energy cascade in the Fourier frequency domain [55]. In this paper, an analysis has performed on the kinetic energy cascade rates at the full spectrum (*f* > 10) and large-scale coherent structures (*f* < 10).

The simulations were performed at Reynolds numbers Re = 100, 400 and 600. Eight points were selected to quantitatively differentiate between the Newtonian and non-Newtonian viscosity models. P1 is located at the center of the bifurcation while P5 and P6 are located at the center of the daughter vessels. P2, P7 and P8 are located near the apex of the bifurcation, while P3 and P4 are located at the heels of the daughter vessels as shown in Fig 1B. The quantitative comparison of the Newtonian model versus the non-Newtonian model in the ICA showed the differences between both models and the impact of using the non-Newtonian on predicting the energy spectra.

The spectra whose slope was high were used to compute the dissipation rate for that axial location and time and indicated the flow instabilities, because energy is transferred from a fundamental frequency to its subharharmonic. Such a process was done when the large vortices broke down to small vortices and as slope increases the kinetic energy cascades at the large-scale coherence more quickly through vortex motions of smaller and smaller scales until it is converted into thermal energy. Transfer of energy from the mean flow to the turbulence occurs mainly at the coherent structures. A coherent structure is a large-scale vortex structure which preserves its spatial and temporal characteristics during a long time. The coherent structures form the lynchpin of studying the flow stability. Coherent structures control the momentum transfer at the larger scale of the energy cascade towards dissipation. It appears evident that these coherent structures established as a result of flow instabilities in the near wall region [75]. when the flow accelerates to the peak flow rate, these coherent structures were moved from the near wall into the core flow [76]. To the best of the authors’ knowledge, the illustrated slopes of the kinetic energy cascade in the present work do not coincide any of the Kolmogorov scales stated in literature. Therefore, the turbulent flow, in this case, does not fall within the Kokmogrov theory and is of non-Kolmogorov turbulence.

#### 3.1.1. Full spectrum kinetic energy cascade rate of the ICA bifurcation

**Fig 3:**
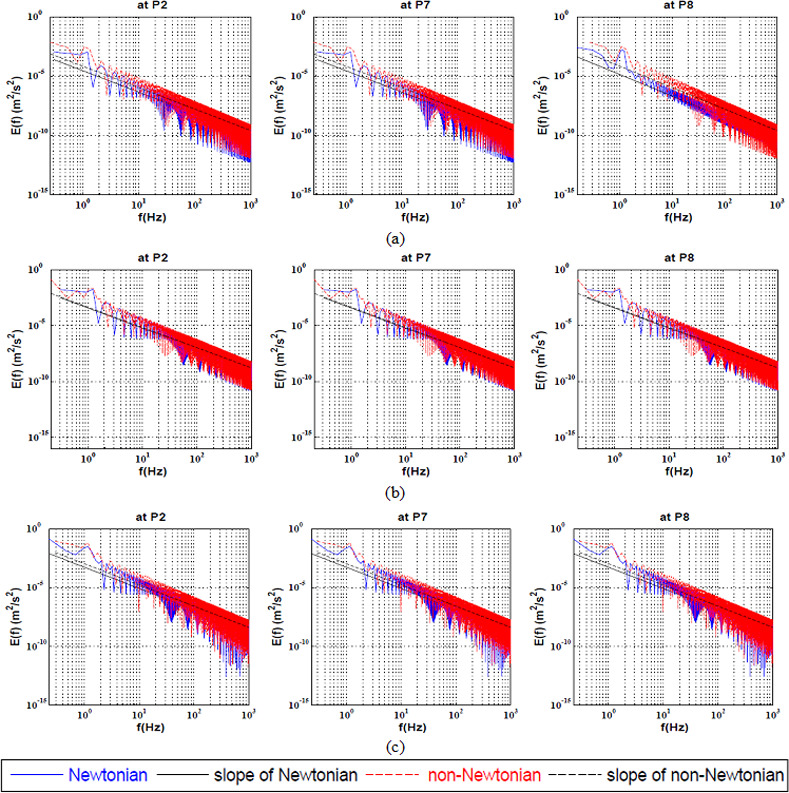
Full Spectrum Energy cascade for Newtonian and non-Newtonian at three points near the apex of the bifurcation artery at a) Re = 100, b) Re = 400 and c) Re = 600.

The full spectrum KEC rates near the apex of the bifurcation at Reynolds ranging from 100 to 600 is presented in (Fig 3). It is clear that the values of energy cascade slope are different from such commonly observed in the classical theory of turbulence.

It was observed that the difference between the Newtonian and Power-Law model at Re = 100 was approximately $≅\;\frac{1}{30}$. However, the energy decay does not change for each model for the whole spectrum. In addition, it was found that by increasing the Reynolds number to 400 and further to 600, the KEC rates remains unchanged for both rheological models and the effect of the shear-thinning on the energy cascade disappears as shown in Table 3.

**Table 3:** The values of the full spectrum KEC rates for power-law model in comparison with Newtonian model at the apex of the bifurcation.

Location	Rheology	Re = 100	Re-400	Re = 600
**P2**	**Newtonian**	-1.81	-1.8	-1.8
	**Power-law**	-1.8	-1.8	-1.8
**P7**	**Newtonian**	-1.81	-1.8	-1.8
	**Power-law**	-1.8	-1.8	-1.8
**P8**	**Newtonian**	-1.81	-1.8	-1.8
	**Power-law**	-1.8	-1.8	-1.8

**Fig 4:**
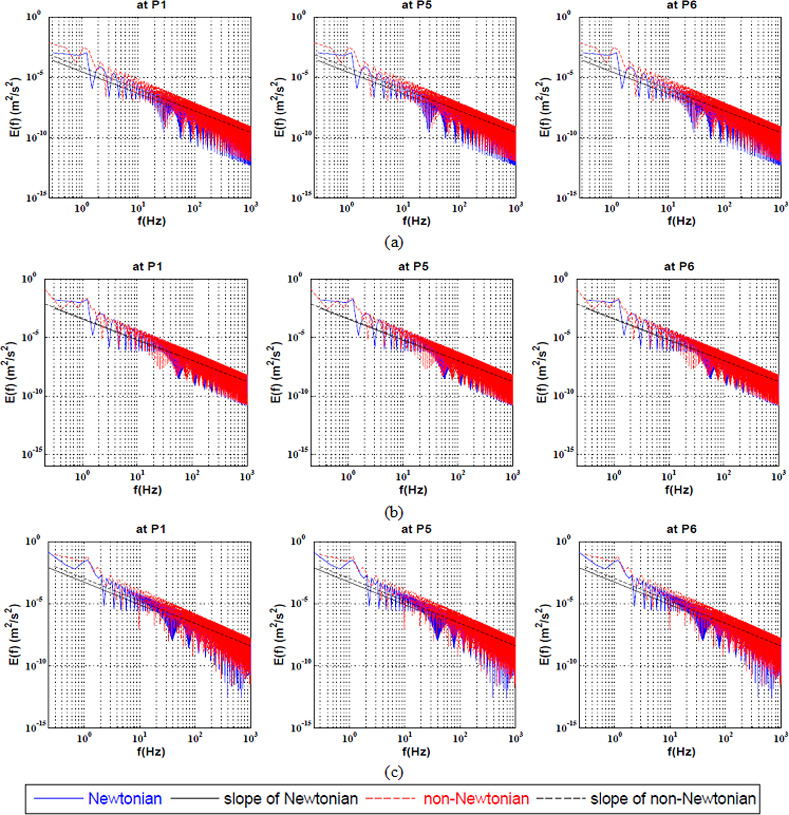
Full Spectrum Energy cascade for Newtonian and non-Newtonian at three points at the center of the bifurcation artery at a) Re = 100, b) Re = 400 and c) Re = 600.

The full spectrum KEC rates at the center of the bifurcation was investigated qualitatively as shown in (Fig 4) and quantitatively as shown in Table 4. It was found that the non-Newtonian had a stabilizing effect at Re = 100. Furthermore, it was observed that by increasing the Reynolds number, both rheological models were similar.

**Table 4:** The values of the full spectrum KEC rates for power-law model in comparison with Newtonian model at the center of the bifurcation.

Location	Rheology	Re = 100	Re-400	Re = 600
**P1**	**Newtonian**	-1.81	-1.8	-1.8
	**Power-law**	-1.8	-1.8	-1.8
**P5**	**Newtonian**	-1.81	-1.8	-1.8
	**Power-law**	-1.8	-1.8	-1.8
**P6**	**Newtonian**	-1.81	-1.8	-1.8
	**Power-law**	-1.8	-1.8	-1.8

(Fig 5) illustrates the full spectrum kinetic energy decay of the power-law model in comparison with the Newtonian model at the heels of the bifurcation artery. It was shown that the non-Newtonian model stabilized the flow at the heels of the bifurcation at the low Reynolds number. By increasing the Reynolds to 400 and 600, it was found that the shear-thinning effect on the full spectrum KEC vanished as shown in Table 5.

**Fig 5:**
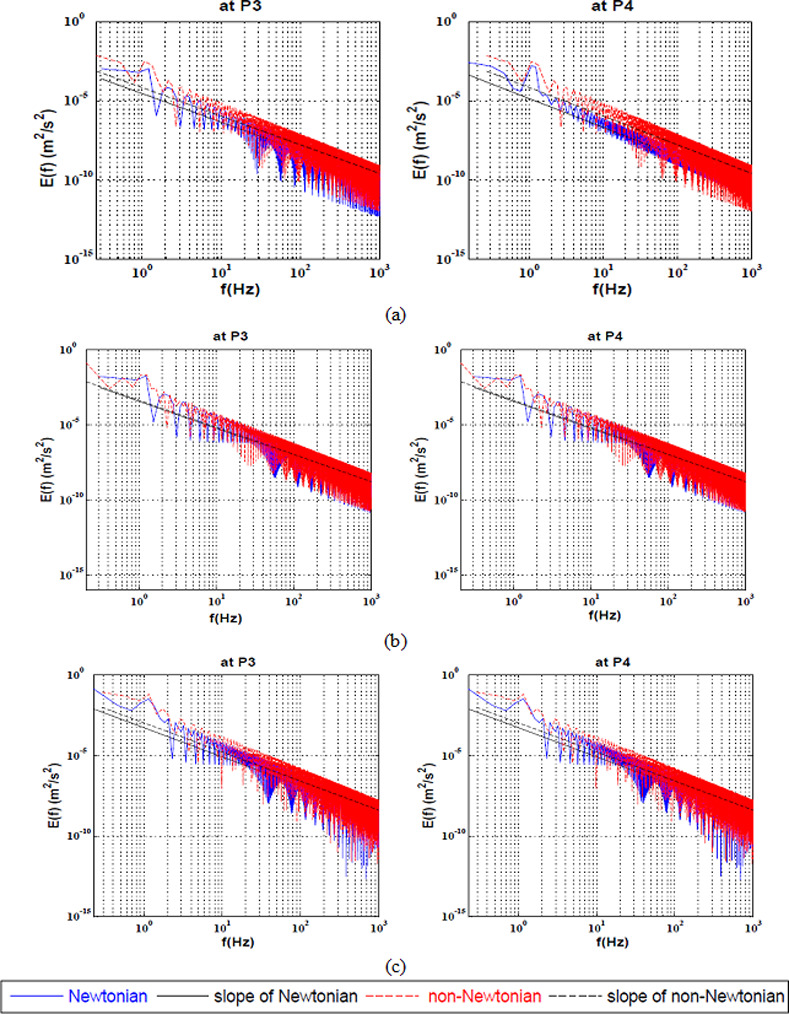
Full Spectrum Energy cascade for Newtonian and non-Newtonian at two points near the heels of the bifurcation artery at a) Re = 100, b) Re = 400 and c) Re = 600.

**Table 5:** The values of the full spectrum KEC rates for power-law model in comparison with Newtonian model at the heels of the bifurcation.

Location	Rheology	Re = 100	Re-400	Re = 600
**P3**	**Newtonian**	-1.81	-1.8	-1.8
	**Power-law**	-1.8	-1.8	-1.8
**P4**	**Newtonian**	-1.81	-1.8	-1.8
	**Power-law**	-1.8	-1.8	-1.8

#### 3.1.2. Kinetic energy cascade rate at the large-coherent structure of the ICA bifurcation

**Fig 6:**
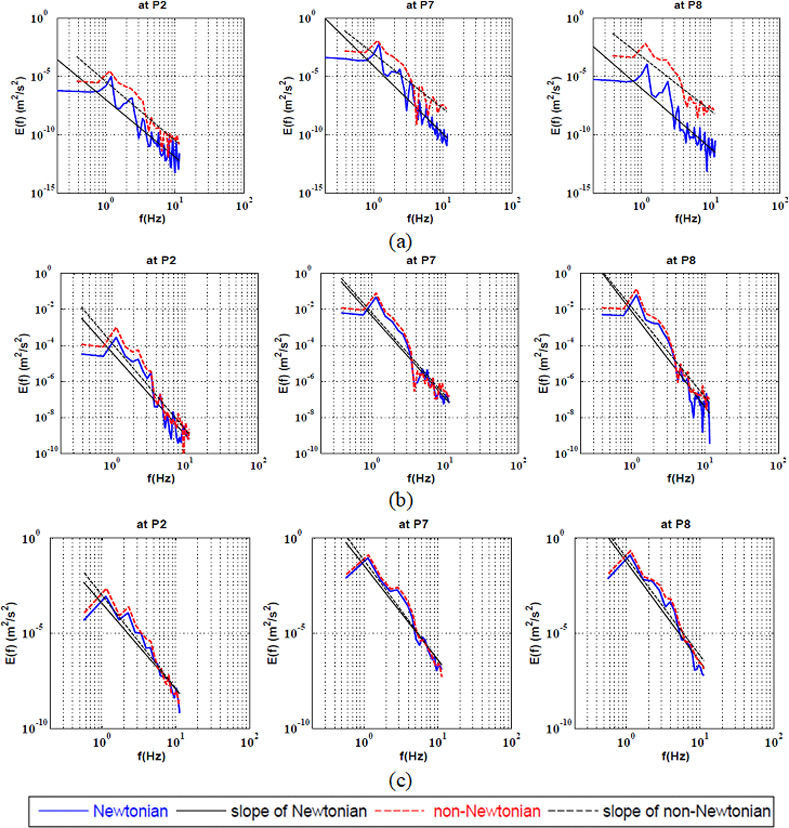
Energy cascade at the large-coherent structure for Newtonian and non-Newtonian at three points near the apex of the bifurcation artery at a) Re = 100, b) Re = 400 and c) Re = 600.

The KEC rates at the large-coherent structure near the apex of the bifurcation at Reynolds ranging from 100 to 600 is presented in (Fig 6).

It can be said that the non-Newtonian model had a destabilizing effect at the apex of the bifurcation P2 at all values of Reynolds number. This explains, beyond the consensual wall shear stress (WSS) theory, why aneurysm mostly initiates at the bifurcation apex and curvatures. Studies revealed that the essential factors in several vascular disorders such as intracranial aneurysms have related to the endothelial dysfunction (ED) and degeneration of the internal elastic lumina (IEL) [8, 34, 77]. In addition, it was found that the blood flow instabilities in arteries are the main reason for endothelial cell dysfunction leading to aneurysm initiation [34]. In-vitro pathological examinations of the ED, IEL degeneration and loss of smooth muscle cells have demonstrated that they are mainly caused and developed by the flow instabilities and the transition to turbulent flow [34, 78]. Once the endothelial cell was damaged, subsequent remodelling and degradation in the vessel wall may occur. The results illustrated that the Newtonian model remarkably underestimated the blood flow instabilities and stabilized the flow. In this sense, the non-Newtonian behavior cannot be ignored as it destabilizes the blood flow which in turn will promote endothelial cell pro-inflammatory pathways, matrix degeneration and loss leading to IA genesis. In addition, the non-Newtonian model had a destabilizing effect at P7 only at Re = 400. However, by increasing the Reynolds number to 600, the non-Newtonian model stabilized the flow. In addition, it was found that at P8, the non-Newtonian model had a stabilized effect on the flow as shown in Table 6.

**Table 6:** The values of the KEC rates for power-law model in comparison with Newtonian model at the large-coherent structure at the apex of the bifurcation.

Location	Rheology	Re = 100	Re-400	Re = 600
**P2**	**Newtonian**	-4.9	-4.6	-4.5
	**Power-law**	-5.1	-4.8	-4.6
**P7**	**Newtonian**	-5.8	-4.5	-5
	**Power-law**	-4.7	-4.6	-4.8
**P8**	**Newtonian**	-5.1	-5.3	-5.3
	**Power-law**	-4.7	-5	-4.9

**Fig 7:**
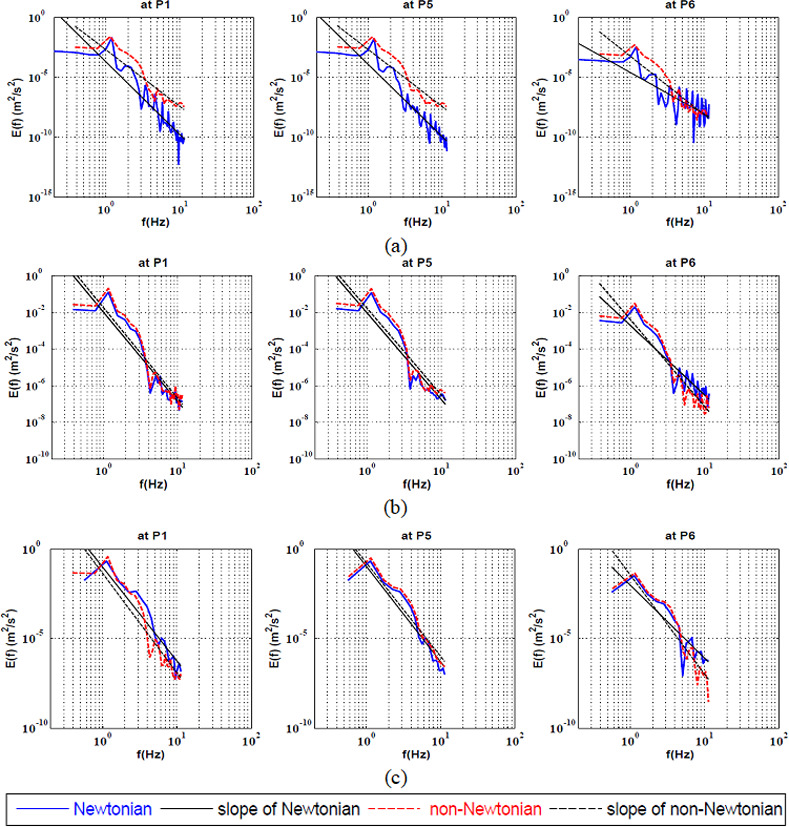
Energy cascade at the large-coherent structure for Newtonian and non-Newtonian at three points at the center of the bifurcation artery at a) Re = 100, b) Re = 400 and c) Re = 600.

The kinetic energy cascade at the center of the bifurcation artery was investigated qualitatively as shown in (Fig 7) and quantitatively as shown in Table 7. It was found that the non-Newtonian model had a destabilizing effect at all values of Reynolds number at P6 which located at separation point of the small daughter vessel. In addition, it was observed that the energy cascade of both rheological models had similar behaviour at P1 and P5. It was found that, the power law model stabilized the flow at low Reynolds number. On the other hand, by increasing the Reynolds number to 400, it was found that the shear thinning effect destabilized the flow compared to such Newtonian flow.

**Table 7:** The values of the KEC rates for power-law model in comparison with Newtonian model at the large-coherent structure at the center of the bifurcation.

Location	Rheology	Re = 100	Re-400	Re = 600
**P1**	**Newtonian**	-6	-4.8	-5.2
	**Power-law**	-4.7	-5	-4.9
**P5**	**Newtonian**	-6	-4.73	-5.3
	**Power-law**	-4.8	-4.74	-5
**P6**	**Newtonian**	-3.5	-3.8	-4.1
	**Power-law**	-4.9	-4.7	-4.8

(Fig 8) illustrates the energy decay of the power-law model comparing to the Newtonian model at the heels of the bifurcation artery. It was shown that the non-Newtonian model destabilized the flow at the heels of the bifurcation at the low Reynolds number. By increasing the Reynolds to 400, it was found that the shear-thinning had a stabilizing effect on the flow pattern. Then, by further increasing Reynolds to 600, the shear-thinning destabilized the flow again as shown in Table 8.

**Fig 8:**
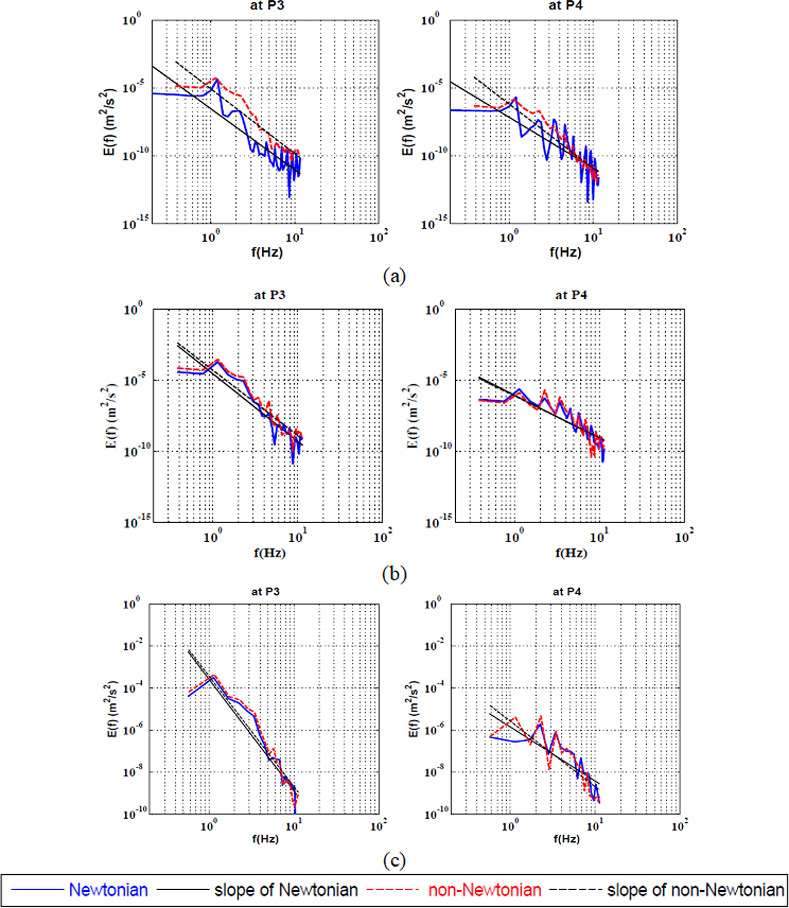
Energy cascade at the large-coherent structure for Newtonian and non-Newtonian at two points at the heels of the bifurcation artery at a) Re = 100, b) Re = 400 and c) Re = 600.

**Table 8:** The values of the KEC rates for power-law model in comparison with Newtonian model at the large-coherent structure at the heels of the bifurcation.

Location	Rheology	Re = 100	Re-400	Re = 600
**P3**	**Newtonian**	-4.5	-4.8	-3
	**Power-law**	-5	-4.6	-3.1
**P4**	**Newtonian**	-4.1	-3	-2.6
	**Power-law**	-5	-2.93	-3.1

The existence of non-Kolmogorov turbulence in blood flow in ideal cerebral artery as shown in Figs 3–8. Near-wall instabilities could be significant and influential in the ECs mechano-signaling promoting the vessel wall degradation leading to aneurysm initiation. By studying the spectral analysis and analysing the KEC, it was found that at the apex of the bifurcation model where aneurysm mostly initiates; the non-Newtonian model had a destabilizing effect at all values of Reynolds number. Consequently, one can argue that the Newtonian assumption underestimate the flow instability. These results could influence the physical explanation of the actual IA hemodynamics.

### 3.2 Vortex structure

**Fig 9:**
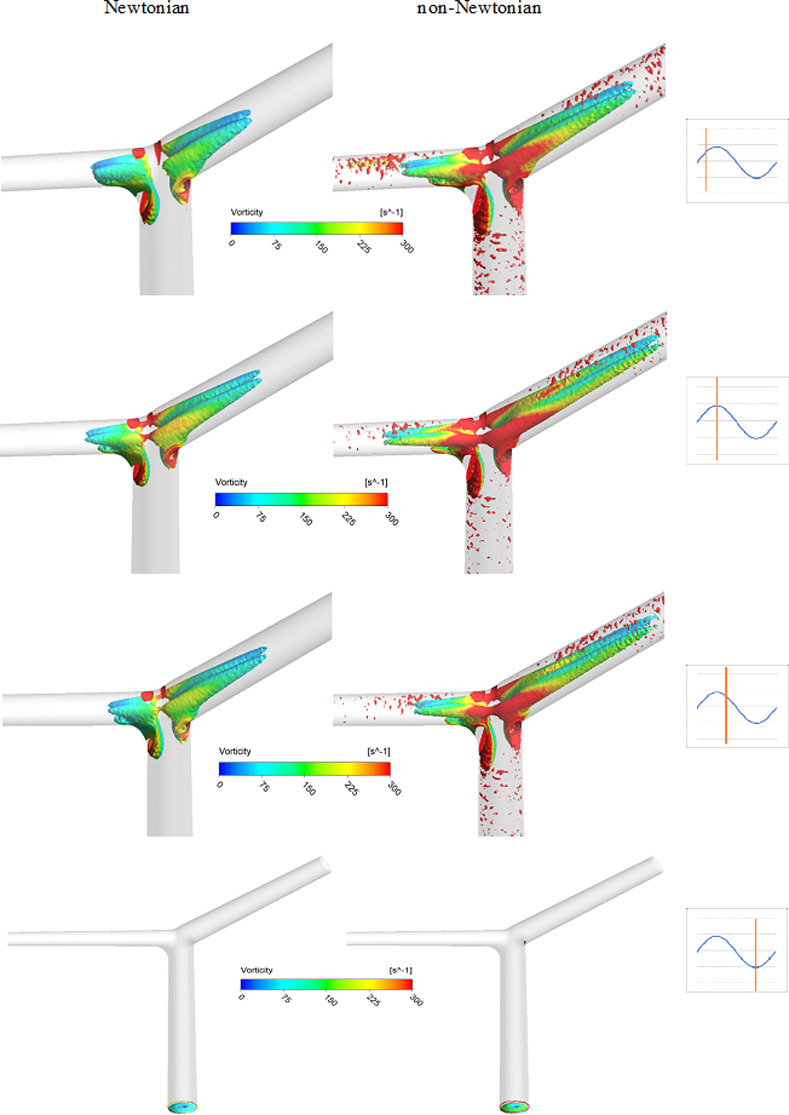
Comparison of identified vortex cores between Newtonian and non-Newtonian models inside the bifurcation model at Re = 100. The marked waveform shows the data point in the cardiac cycle used for extracting the structures. The vortex visualization using the Q-criteria at threshold (0.05). Colored with the vorticity.

The vortex-identification inside the internal carotid bifurcation artery has been studied for Newtonian and non-Newtonian viscosity models based on the Q-criterion which used to describe and identify the vortex regions. The volume of the region occupied by vortex structures has been visualized as shown in (Fig 9). The simulations were performed at Reynolds number = 100. The vortex patterns for both viscosity models were depicted at an arbitrary threshold to see the coherence vortex structures and at four-time instants along the cardiac cycle: mid-acceleration, peak systole, mid-deceleration and peak diastole respectively.

**Fig 10:**
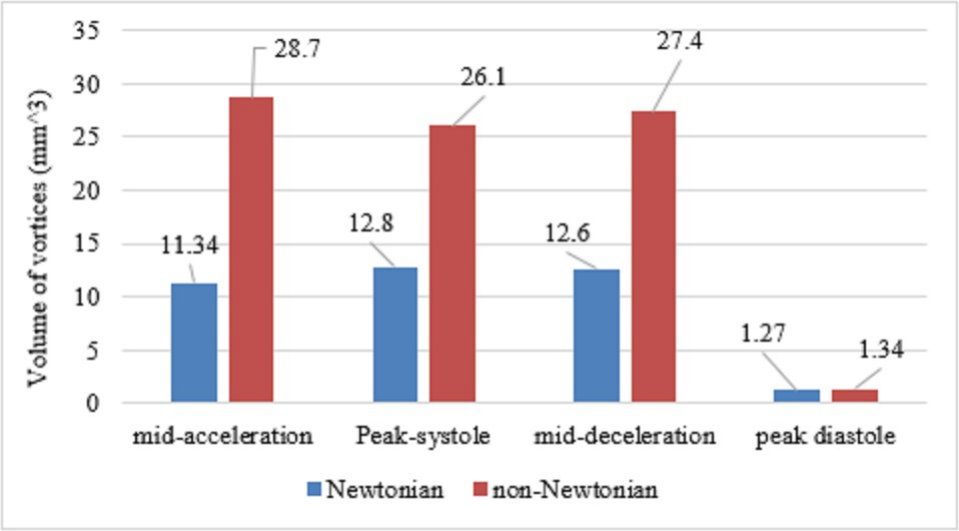
The results of the size of the coherence vortex structures of the Newtonian model in comparison with the non-Newtonian model at Re = 100 in the ICA at four-time instants using Q criteria.

It was found that the vortex size of the non-Newtonian model was higher than the Newtonian at all-time instants as shown in Fig 9 by using the Q-criterion. In addition, it was observed that the volume of the vortex structures of the Newtonian viscosity model remained unchanged and localized at the apex of the bifurcation, while the volume of the dean vortices of the non- Newtonian viscosity model varied during the cardiac cycle.

(Fig 10) represents the variations of identified vortex volumes quantitatively at Re = 100 due to the selection of two different viscosity models at four-time instants. Based on the Q-criteria method, it was found that the volume of the zone occupied by coherence vortical structures of the non-Newtonian model was approximately 2.5 times the Newtonian model at the mid-acceleration. While this difference decreased to be 2 times at the peak systole and then increased again to 2.2 times at the mid-deceleration due to the inertia force which was dominating the flow. Finally, there was no difference between the two viscosity models at the diastolic peak.

The vortex structures inside the ICA based on the Q-criterion have been visualized and quantified at Reynolds number = 100. The impact of shear-thinning fluid on the size of the coherent vortex structure compared to the Newtonian model has been investigated. It was observed that the size of the vortex of the non-Newtonian model was higher than the Newtonian model at the four-time instants, and these observed differences could not be ignored.

## 4. Conclusion

This study challenges a commonly used assumption in modeling blood in intracranial arteries. Direct Numerical Simulation (DNS) was conducted to investigate the impact of non-Newtonian blood behavior in an idealized ICA bifurcation. The incidence of transitional flow at low Reynolds number of the order of 100, 400 and 600 has been investigated. The kinetic energy cascade rates and the vortex structure suggest substantial differences exist between the two viscosity models. These differences highlight the demand for further investigations that can integrate realistic flow characteristics and patient-specific geometries. From the proposed comparative analysis, the authors suggest that the non-Newtonian viscosity model should be taken into consideration while simulating blood flow in intracranial arteries as it affects the stability of the blood flow which in turn will exhibit endothelial dysfunction leading to many cerebrovascular lesions as intracranial aneurysms.

## Supporting information

S1 Nomenclature (DOCX)
