# Supplementary material for: Numerical study on the energy cascade of pulsatile Newtonian and power-law flow models in an ICA bifurcation
Source: PLoS One. 2021 Jan 25;16(1):e0245775. doi: 10.1371/journal.pone.0245775 (PMC7833255; doi:10.1371/journal.pone.0245775)
Supplement: S1 Nomenclature — (DOCX) [file pone.0245775.s001.docx]

**S1 Nomenclature.**

| \| $\rho$ Density of the fluid  $\mathbf{v}$ Velocity vector field  $p$ Pressure  $\mu$ Dynamic viscosity  $\upsilon$ Kinematic viscosity  $k$ Flow consistency index  $n$ Power law index  $\dot{\gamma}$ Shear rate  $\boldsymbol{\tau}$ Stress tensor  $\mathrm{Re}$ Reynolds number  C Courant number  $\Delta t$Time-step of the numerical model  $\Delta x$ Spacing of the grid in the numerical model \| $u_{m}$ mean velocity  R radius of the artery  d diameter of the artery  r radial direction  $u_{os}$ Amplitude of the oscillating component (sine wave)  $f$ frequency of oscillation  $\omega$ angular frequency  $p$ pressure  KEC Kinetic energy cascade  $u\left( r,t \right)$ axial velocity variation with time and radial position  DNS Direct numerical simulation  ICA Internal carotid artery \| \| --- \| --- \| |
| --- | --- | --- |
